# Bariatric surgery for diabetic comorbidities: A focus on hepatic, cardiac and renal fibrosis

**DOI:** 10.3389/fphar.2022.1016635

**Published:** 2022-10-21

**Authors:** Huanxin Ding, Yun Zhang, Xiaomin Ma, Zhongwen Zhang, Qian Xu, Chuxuan Liu, Bingjun Li, Shuohui Dong, Linchuan Li, Jiankang Zhu, Mingwei Zhong, Guangyong Zhang

**Affiliations:** ^1^ Department of General Surgery, Shandong Provincial Qianfoshan Hospital, Shandong University, Jinan, Shandong, China; ^2^ Department of General Surgery, The First Affiliated Hospital of Shandong First Medical University, Jinan, Shandong, China; ^3^ Department of Endocrinology, The First Affiliated Hospital of Shandong First Medical University, Jinan, Shandong, China

**Keywords:** bariatric surgery, Roux-en-Y gastric bypass, sleeve gastrectomy, diabetic cardiomyopathy, non-alcoholic fatty liver disease, diabetic kidney disease, fibrosis

## Abstract

Continuously rising trends in diabetes render this disease spectrum an epidemic proportion worldwide. As the disease progresses, the pathological effects of diabetes may impair the normal function of several vital organs, eventually leading to increase the risk of other diabetic comorbidities with advanced fibrosis such as non-alcoholic fatty liver disease, diabetic cardiomyopathy, and diabetic kidney disease. Currently, lifestyle changes and drug therapies of hypoglycemic and lipid-lowering are effective in improving multi-organ function, but therapeutic efficacy is difficult to maintain due to poor compliance and drug reactions. Bariatric surgery, including sleeve gastrectomy and Roux-en-Y gastric bypass surgery, has shown better results in terms of prognosis for diabetes through long-term follow-up. Moreover, bariatric surgery has significant long-term benefits on the function of the heart, liver, kidneys, and other organs through mechanisms associated with reversal of tissue fibrosis. The aim of this review is to describe the impact of type 2 diabetes mellitus on hepatic, cardiac and renal fibrosis and to summarize the potential mechanisms by which bariatric surgery improves multiple organ function, particularly reversal of fibrosis.

## Graphical Abstract

**Figure:**
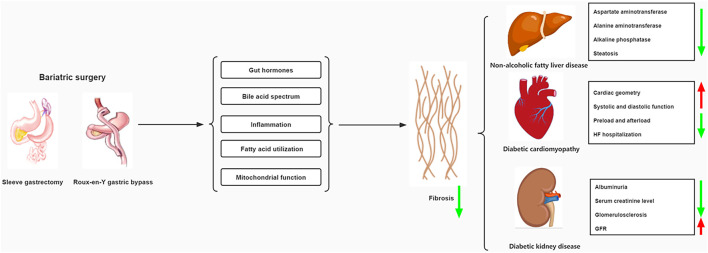
Pharmacological treatment and mechanisms of bariatric surgery for diabetic comorbidities.

## 1 Introduction

As a global prevalent metabolic disease, diabetes mellitus (DM) is characterized by a lack of insulin production or cellular uptake of insulin (2018). Insulin is essential for controlling glucose levels in the body (Tokarz et al., 2018). In type 2 diabetes mellitus (T2DM), insulin resistance (IR) affects the metabolism of carbohydrates, proteins and lipids, thereby promoting the development of hyperglycemia and hyperlipidemia (Taylor, 2013).

The state of being chronically overweight combined with T2DM substantially increases the incidence of comorbidities such as non-alcoholic fatty liver disease (NAFLD), diabetic cardiomyopathy (DCM) and diabetic kidney disease (DKD) (Al-Sulaiti et al., 2019; La Sala and Pontiroli, 2020). As the degree of organ fibrosis increases, the functions of the liver, heart and kidney are gradually damaged and become more and more serious. The fibrotic process of these diseases has been slowed down to some extent by conservative medical management such as pharmacological interventions and lifestyle changes (Cox et al., 2013). However, low adherence makes it difficult to maintain positive treatment outcomes.

Over the past few decades, the role of bariatric surgery for long-term improvement in metabolic disease has received increasing attention (Hanipah and Schauer, 2020). There is substantial evidence that surgery is better at improving weight loss and T2DM outcomes than nonsurgical interventions, regardless of the type of surgery used. The 1991 National Institutes of Health guidelines recommend consideration of bariatric surgery in patients with a body mass index [BMI (calculated as weight in kilograms divided by height in meters squared)] of at least 40 kg or at least 35 kg with severe obesity-related comorbidities (1991). However, there is growing evidence that bariatric surgery should also be considered in people with T2DM with a BMI of 30–35 kg who are not adequately controlling hyperglycemia despite optimal T2DM medication Operation (Di Lorenzo et al., 2020). As the most commonly used bariatric surgery, Roux-en-Y gastric bypass (RYGB)and Sleeve gastrectomy (SG)not only have significant effect on weight loss and hypoglycemia, but also can improve or even reverse the complications of T2DM in the long term (Arterburn et al., 2020). Therefore, we summarize the effects of treating T2DM on fibrosis and the current status of bariatric surgery, with a focus on the underlying mechanisms by which bariatric surgery improves function and fibrosis of vital organs. This provides evidence that bariatric surgery may be a better treatment for these conditions.

## 2 Effects of type 2 diabetes mellitus on fibrosis of vital organs

Acute and chronic inflammation often triggers fibrosis in various vital organs (Mack, 2018; Henderson et al., 2020). Inflammation results in damage to resident epithelial cells and often endothelial cells, leading to increased release of inflammatory mediators including cytokines, chemokines, etc. This process contributes to the recruitment of numerous inflammatory cells, including lymphocytes, polymorphonuclear leukocytes, eosinophils, basophils, mast cells, and macrophages. These inflammatory cells trigger the activation of effector cells that drive the process of fibrosis (Wynn, 2004). Fibroblasts and myofibroblasts serve as key fibrotic effectors in several organs and are responsible for the synthesis of extracellular matrix (ECM) proteins (Hinz et al., 2007). The matrix proteins that make up fibrotic scars are mainly composed of interstitial collagens (types I and III), cellular fibronectin, and basement membrane proteins (Rockey et al., 1993). Molecular systems involved in fibrosis include platelet-derived growth factor (PDGF), connective tissue growth factor (CTGF), and the vasoactive peptide system, especially angiotensin II and endothelin-1 (Wynn, 2007).

Liver fibrosis is usually caused by an inflammatory process resulting in injuries to liver cells or bile duct cells. Inflammation leads to the activation of effector cells, which contributes to the deposition of ECM (Bessone et al., 2019). Although multiple effectors synthesize ECM in the liver, hepatic stellate cells (HSCs) appear to be the major source of ECM. There is substantial evidence that hepatic stellate cells transform into myofibroblasts upon injury (Rockey et al., 1993). IR worsens with NAFLD disease progression and is considered a component of the pathogenesis of liver fibrosis (Friedman et al., 2018; Schwabe et al., 2020). One study showed that IR-related hyperinsulinemia directly stimulates HSCs proliferation and type I collagen secretion by activating PI3K and ERK-dependent pathways (Svegliati-Baroni et al., 1999). Inflammation can be caused by IR itself. Simultaneously, hepatocyte stress and death also promote inflammation, leading to macrophage recruitment and secretion of fibrotic-derived mediators, such as transforming growth factor beta (TGF-β), which is central to the liver fibrotic response (Koyama and Brenner, 2017; Tsuchida and Friedman, 2017).

Cardiomyocyte hypertrophy and excessive deposition of ECM are pivotal to the cardiac remodeling after injury (Porter and Turner, 2009). Cardiac fibrosis is generally divided into two types including reactive fibrosis and replacement fibrosis (Liu et al., 2021). Reactive fibrosis occurs in perivascular spaces. Replacement fibrosis occurs at the site of muscle cell loss. Fibrosis in the heart is attributed to fibroblasts, which are the most abundant cell type in the heart muscle. These cells are derived from cardiomyocyte-native fibroblasts, circulating fibroblasts, and fibroblasts resulting from the epithelial-mesenchymal transition (Zeisberg et al., 2007; Moore-Morris et al., 2014). The process by which all these cell types proliferate and differentiate into myofibroblasts after injury is driven by factors such as TGF-β, endothelin-1, and angiotensin II (Ang II). Type I and III interstitial collagen deposition was significantly increased in DCM patients compared with myocardial biopsies in non-diabetic patients, corroborating reports of cardiac interstitial and perivascular fibrosis found at earlier autopsy (van Hoeven and Factor, 1990; Shimizu et al., 1993). It has also been suggested that an important component of the increased cardiac fibrosis caused by T2DM may be replacement fibrosis. The above indicates that the two types of fibrosis are likely to coexist in DCM (Regan et al., 1977).

T2DM and hypertension are two major causes of renal fibrosis (Kaissling et al., 2013). Renal fibrosis is mediated by cellular components (e.g., inflammatory cells) and molecular components (e.g., cytokines, TGF-β, and endothelin-1) (Liu, 2011). The renin-angiotensin-aldosterone axis is particularly important in hypertension-induced fibrosis (Mezzano et al., 2001). The kidney has a unique cellular structure consisting of glomeruli, tubules, interstitium and capillaries. Damage to any of these sites triggers the deposition of ECM. Abnormal and excessive deposition of ECM proteins in the glomerular and interstitial regions are typical features of renal fibrosis (Sun et al., 2016). In DKD, T2DM and systemic hypertension exacerbate glomerular capillary pressure, leading to proteinuria, activation of cytokines and complement, and infiltration of immune cells, ultimately resulting in epithelial and interstitial fibrosis (Liu, 2011).

## 3 Non-alcoholic fatty liver disease

A large number of diabetic patients develop NAFLD and non-alcoholic steatohepatitis (NASH). NAFLD is the most common cause of chronic liver disease and includes a series of liver diseases ranging from simple steatosis to NASH, fibrosis and cirrhosis (Powell et al., 2021). The incidence of NAFLD is rising due to the diabetes pandemic, with a global prevalence of 24% in the general population (Younossi et al., 2018). NASH is an inflammatory subtype of NAFLD with evidence of steatosis and hepatocellular damage (ballooning) and inflammation, with or without fibrosis (Chalasani et al., 2018). Regardless of the underlying mechanism, significant weight loss is known to have beneficial effects on NASH, with sustained weight loss of 10% being associated with reduced liver fibrosis (Vilar-Gomez et al., 2015). An ideal treatment would effectively reverse liver damage and fibrosis without negatively affecting other metabolic parameters or cardiovascular complications.

### 3.1 Effects and underlying mechanisms of bariatric surgery in improving non-alcoholic fatty liver disease

Bariatric surgery can improve liver metabolism and reduce biochemical indicators of nonalcoholic fatty liver disease, including aspartate aminotransferase, alanine aminotransferase, and alkaline phosphatase, as well as histological parameters such as steatosis, fibrosis, Lobular inflammation and hepatocyte ballooning (Bower et al., 2015). Reduction in hepatic fat content and improvement in hepatic insulin resistance is one of the earliest beneficial effects of bariatric surgery and thus constitutes an attractive treatment option for NAFLD (Fakhry et al., 2019). General retrospective or cohort studies suggest that improvement in NAFLD after bariatric surgery is more likely than with other interventions; however, current data are insufficient to demonstrate a reduction in liver-specific mortality (Wolfe et al., 2016). Among patients with biopsy-proven NAFLD/NASH, the majority of SG and RYGB patients had essentially normal liver function in the 1-year follow-up, and the two procedures were equally effective in improving liver function (Cherla et al., 2020; de Brito et al., 2021). In addition, NAFLD patients underwent RYGB were more likely to experience early transient deterioration of liver function than those underwent SG, possibly due to postoperative malabsorption or rapid weight loss (Kalinowski et al., 2017). However, RYGB seems to have more advantages in improving hepatic fibrosis (de Brito et al., 2021). Liver biopsies from 180 severely obese patients with biopsy-proven NASH after bariatric surgery showed that 84% of patients observed regression of NASH at 1 year after bariatric surgery, with no apparent recurrence within 1–5 years (*p* = 0.17). Fibrosis began to decrease 1 year after surgery and continued to decrease within 5 years (Lassailly et al., 2020).

#### 3.1.1 Effects of altered gut hormone levels

GLP-1 is a peptide hormone and neurotransmitter with many metabolic and non-metabolic effects, secreted from the gut by L cells (Drucker, 2018). Increased GLP-1 levels improve beta cell function and insulin sensitivity (Lima et al., 2013). Elevated postprandial GLP-1 levels in patients after SG or RYGB (Jiménez et al., 2013; Jiménez et al., 2014). While most research on GLP-1 has focused on glucose metabolism, GLP-1 functions beyond glycemic control. GLP-1 promotes satiety, enhances insulin release, and inhibits glucagon release in response to nutrient intake (Jiménez et al., 2014). These findings point to possible mechanisms of relative glycemic control and weight loss found after surgery involving GLP-1, glucose homeostasis and T2DM remission. In NASH patients, GLP-1 reduces *de novo* lipogenesis, lipolysis-induced free fatty acid levels, and triglyceride-derived toxic metabolites (Armstrong et al., 2016b). In animal experiments comparing the therapeutic effects of liraglutide and bariatric surgery, bariatric surgery may enhance GLP-1 secretion by increasing serum bile acids and L cell proliferation in the ileum compared with liraglutide, thereby Ameliorates hepatic steatosis in obese rats (Kashihara et al., 2015). In addition, in a phase 2 trial on NAFLD, liraglutide resulted in some resolution of liver fibrosis (Armstrong et al., 2016a). On this basis, bariatric surgery appears to be more likely to alleviate the degree of liver fibrosis through a GLP-1-mediated mechanism.

In humans, hormone-like members of the FGF family include FGF-15/19 and FGF-21 (Itoh, 2010). The metabolism of FGF-19 and FGF-21 is involved in the regulation of bile acid biosynthesis, glucose metabolism and lipid metabolism in beta cells of pancreatic islets, liver and adipose tissue (Fisher et al., 2011). FGF-15 and FGF-21 have been shown to be protective against liver fibrosis in a mouse model (Zarei et al., 2018; Liu et al., 2020). Plasma FGF-19 and FGF-21 levels were analyzed in 28 patients who received RYGB (Harris et al., 2017). They found that RYGB surgery resulted in increased postprandial plasma FGF-19 concentrations. They also found that plasma FGF-21 exceeded baseline levels by approximately three times after RYGB surgery. Bariatric surgery induces FGF-15/19 expression in the distal small intestine *via* direct stimulation of FXR by bile acids (Huang et al., 2019; Eiken et al., 2020). Furthermore, FGF-15/19 acts in an endocrine-like manner to improve the metabolic function of insulin target tissues. DJB and SG surgery restores the FGF21 signaling pathway in the liver of high-fat diet/streptomycin-induced diabetic rats and increases FGF21 sensitivity (Liu et al., 2019).

PYY, also known as a short peptide of pancreatic polypeptide YY3-36, is released by L cells in the distal small intestine and colon (le Roux and Bloom, 2005). The main effects of PYY involve the central regulation of appetite as well as the regulation of blood glucose homeostasis (Batterham et al., 2003). In mouse models, PYY modulates weight loss after bypass surgery (Chandarana et al., 2011), while increased PYY has been observed in patients after bariatric surgery, both as a result of post-operative anatomical changes in the gastrointestinal tract (Magouliotis et al., 2017). Notably, several studies suggest that PYY3-36 levels are reduced in obesity and elevated after gastric bypass surgery, possibly contributing to increased satiety after this procedure (Price and Bloom, 2014; Guida et al., 2017). Overall, as PYY is strongly associated with weight loss and NAFLD after bariatric surgery, its role in metabolism warrants further investigation. Although more research is needed, elevated levels of PYY are associated with stabilization of blood glucose levels, metabolism, and body weight, which directly affect the incidence and complications of obesity and T2DM.

#### 3.1.2 Changes in bile acid metabolism, transport and signal transduction

Both RYGB and SG have been shown to induce sustained increases in systemic bile acid (BA) concentrations and intestinal BA signaling, which are associated with improvements in insulin resistance and glycemic control (Belgaumkar et al., 2016; Risstad et al., 2017). Bile acids are signaling molecules and metabolic integrators, which involve in activating nuclear farnesoid X receptor (FXR) and membrane Takeda G-protein-coupled receptor 5 (TGR5) to regulate glucose, lipid and energy metabolism (Chiang and Ferrell, 2020). However, BA includes more than 20 components, each of which may have specific effects on metabolism (de Aguiar Vallim et al., 2013). Notably, 12α-hydroxylated bile acids promote liver fibrosis through TGR5-mediated p38MAPK and ERK1/2 signaling, but these bile acids were significantly reduced after SG (Wang et al., 2017; Xie et al., 2021). RYGB upregulates the expression of hepatic BA targets FXR and peroxisome proliferator-activated receptor-α (PPARα) in severely obese patients, and these changes are accompanied by regression of NASH and T2DM after 1 year (Francque et al., 2015; Mazzini et al., 2019). BA treatment of primary and immortalized human hepatocytes induces FXR and PPARα to downregulate glycolysis and restrict glucose entry into the Krebs cycle, thereby inhibiting fatty acid synthesis from excess glucose (Cai and Sewer, 2013). PPARα activation combined with PPARβ/δ agonists improves steatosis, inflammation and fibrosis in preclinical models of NAFLD, identifying new potential therapeutic areas (Pawlak et al., 2015). Activation of the gut FXR/FGF19 signaling pathway inhibits the key lipogenic enzyme, acetyl-CoA carboxylase, supporting the downregulation of *de novo* fatty acid production by hepatic FXR and indirectly promoting fatty acid oxidation by downregulating the expression of carnitine palmitoyl transferase 1α (CPT1α) serving as a key mitochondrial fatty acid transporter. Recent studies have shown that CPT1α is elevated in HSCs from fibrotic patients and mouse models of fibrosis, and that CPT1α induces activation of these cells leading to fibrosis (Fondevila et al., 2022).

Although studies of the effects of SG on FXR and its downstream targets are lacking in humans, the observed increase in post-operative systemic FGF19 concentrations is consistent with enhanced gut FXR signaling following SG. Likewise, findings in FXR-deficient mouse models receiving SG suggest that FXR is essential to achieve the full metabolic benefits of surgery, including postoperative recovery of glucose tolerance and reduced energy intake (Ryan et al., 2014; Cavin et al., 2016). It is worth mentioning that FXR agonists have emerged as a potential therapy against liver fibrosis, despite the issue of side effects (Li et al., 2021). In addition to the direct effects of FXR signaling on hepatic BA, glucose, and fat metabolism, inhibition of intestinal FXR after RYGB is also associated with upregulation of TGR5 (Zhai et al., 2018), which may contribute to postprandial insulin hormone pancreatic elevation in enteroendocrine L cells after surgery Increased levels of GLP1 and PYY suggest a multifaceted antidiabetic effect (Trabelsi et al., 2015; Li et al., 2019).

#### 3.1.3 Reversing inflammation

In a group of patients undergoing bariatric surgery, the expression of inflammatory genes in subcutaneous and visceral adipose tissue was proportional to the severity of NAFLD histology (Rosso et al., 2019). One year post-surgery, SG improved liver histology, especially the degree of fibrosis, in all NASH patients (Cabré et al., 2019). At the same time, there were significant differences in C-C chemokine receptor type 2 (CCR2) and Tumor necrosis factor-α (TNF-α) in preoperative plasma and liver between NASH patients and non-NASH patients, so it is considered that the improvement of liver fibrosis involves the regulation of oxidative stress and inflammation levels. TNF-α, a key cytokine upregulated in obesity, is mainly produced by macrophages (Kunz et al., 2021). Serum levels of various inflammatory markers correlate with liver inflammation, CD11c+ CD206+ and CCR2+ macrophage expansion in visceral adipose tissue of patients with NASH and/or fibrosis (Wentworth et al., 2010; du Plessis et al., 2015). Transplantation of visceral adipose tissue in obese mice increased hepatic macrophage accumulation and exacerbated steatohepatitis compared with a high cholesterol diet model (Bijnen et al., 2018).

Toll-like receptor 4 (TLR4) is considered to be one of the cutting-edge cross-communicators between gut bacteria and the host regulating inflammatory signaling and energy homeostasis (Sala et al., 2020). A pathway specific to liver fibrosis involves TLR4 and is associated with enhanced TGF-β signaling (Seki et al., 2007). TLR4 is activated on the surface of HSCs by gut bacterial lipopolysaccharides from the gut, triggering cell activation and fibrosis formation, thereby linking fibrosis to the microbiome (Sorribas et al., 2019). TLR4 expression in patients with NAFLD is associated with fibrosis (Vespasiani-Gentilucci et al., 2015). It has been shown that RYGB specifically reduces TLR4 expression in the small and large intestines of C57Blc/6J mice, and that TLR4 and myeloid differentiation factor 8 (MyD88, a key downstream signaling regulators) are required for RYGB-induced metabolic responses (Abu El Haija et al., 2021).

## 4 Diabetic cardiomyopathy

There is a broad link between T2DM and cardiovascular disease (CVD). T2DM causes cardiomyopathy and increases the risk of heart failure (HF) even independent of traditional HF risks, including hypertension (HTN), coronary heart disease (CHD), and valvular heart disease (VHD) (Dillmann, 2019). DCM is characterized by abnormal diastolic relaxation at an early stage (Jia et al., 2018). Numerous pathophysiological abnormalities collectively promote interstitial fibrosis, diastolic dysfunction, and later systolic dysfunction in cardiac tissue, resulting in clinical HF syndrome. A retrospective cohort study of 8,231 people with T2DM showed a 2.5-fold increased risk of HF in people with T2DM (Nichols et al., 2004). In addition, an observational study involving 25,958 men and 22,900 women showed that a 1% increase in HbA1c was associated with an 8% increased risk of HF regardless of blood pressure, obesity, age and the presence of coronary heart disease (Iribarren et al., 2001). Therefore, glycemic control in patients with T2DM is a key mechanism to prevent cardiac dysfunction and HF.

### 4.1 Effects and underlying mechanisms of bariatric surgery in improving diabetic cardiomyopathy

Bariatric surgery significantly improves cardiac geometry, function, providing a unique and effective treatment for patients with DCM and HF (Roth et al., 2020). Studies have shown that the remission rate of high blood pressure is 60%–70% within a year after bariatric surgery (Benaiges et al., 2019). In addition to controlling blood pressure, RYGB and SG are effective in reducing cardiovascular risk and the incidence of sudden macrovascular disease (Fisher et al., 2018; Blanco et al., 2019). In severely obese patients with T2DM, weight loss after bariatric surgery improves left ventricular end diastolic dimension (LVDD) (Elagizi et al., 2018). Notably, many patients with pre-existing cardiovascular disease can reduce or completely discontinue cardiovascular medications following bariatric surgery (English and Williams, 2018). Furthermore, according to a large retrospective analysis, bariatric surgery reduces in-hospital mortality by nearly 50% and length of stay in hospitalized patients with HF (Aleassa et al., 2019).

#### 4.1.1 Reducing heart load and cardiac fatty acid utilization

Preload and afterload, as well as systemic and local energy requirements, are decreased after bariatric surgery due to significant reductions in adipose tissue and mass (Lin et al., 2011). Concentrations of vasoconstrictors (e.g., enkephalinase, angiotensin II, renin, and endothelin-1) decreased significantly (*p* < 0.05) 6 months after RYGB by follow-up. These changes promote beneficial “reverse remodeling,” improve fibrosis and the incidence of ventricular tachycardia (Massare et al., 2010) and reduce the incidence of sudden death (Spinale, 2007). Due to lipid metabolism disorder, excessive fat deposition in the heart can create a lipotoxic environment, and at the same time increase the uptake and utilization rate of fatty acids by the myocardium, thereby impairing cardiac function over time (Labbé et al., 2012; Shulman, 2014). By studying positron emission tomography scans of the hearts of 12 obese subjects with T2DM before and after bariatric surgery, Carreau et al. (2020) found that bariatric surgery reduces cardiac fatty acid utilization and enhances left ventricular function.

#### 4.1.2 Improving mitochondrial function and regulating calcium homeostasis

Both cellular and tissue mitochondrial respiration were increased in patients receiving RYGB or SG compared to preoperatively (Nijhawan et al., 2013). Evidence suggests that in a rat model of DCM, SG restores mitochondrial dysfunction and fragmentation by activating 5′ adenosine monophosphate-activated protein kinase (AMPK) signaling (Li et al., 2022)**.** Myocardial Ca^2+^ transients exhibit significantly higher amplitudes and faster decay after SG and DJB, suggesting that bariatric surgery can promote myocardial Ca^2+^ homeostasis in DCM rats (Huang et al., 2018). The above-mentioned postoperative repair of mitochondrial function and Ca^2+^ homeostasis can improve cardiac structure and cardiac function by reducing myocardial collagen deposition and fibrosis (Huang et al., 2018; Li et al., 2022). Despite this, there are currently few clinical studies on specific improvements in postoperative cardiac mitochondrial function and calcium homeostasis in patients undergoing bariatric surgery (Kindel and Strande, 2018).

#### 4.1.3 Adjusting the enterocardiac axis and inhibiting inflammation

Other beneficial effects of bariatric surgery include altered gastrointestinal microbiota, changed bile acid profile, and increased postprandial GLP-1 in the days following surgery (Chambers et al., 2011; Ryan et al., 2014). Bariatric surgery may beneficially alter the enterocardiac axis through one or more of these mechanisms to further improve cardiac function beyond the effects of weight loss. Gut-secreted GLP-1 in cardiomyocytes is now known to lead to decreased apoptosis and increased glucose uptake, and to reverse myocardial fibrosis, independent of canonical insulin signaling (Bose et al., 2005; Helmstädter et al., 2020). Increased level of GLP-1 after bariatric surgery can reverse endothelial dysfunction and restore the endothelial protective properties of high-density lipoprotein (HDL) (Osto et al., 2015). In addition, GLP-1R agonists have been used to treat ischemia-reperfusion injury and HF.

Adiponectin (APN), the most abundant product of white adipose tissue (WAT), has been proposed as a potential prognostic biomarker and therapeutic target in patients with cardiometabolic disease, which is inversely associated with obesity (Messina et al., 2018). Adiponectin levels are associated with a reduction in total fat mass and atherosclerosis risk (Umeda et al., 2013) and are significantly elevated after bariatric surgery (Valenzano et al., 2020). Current studies have shown that APN reduces the degree of cardiac fibrosis by activating autophagy in macrophages and inhibiting Ang II-induced inflammatory responses (Qi et al., 2014). Serum Hsp60 (a kind of adipokine) was elevated in patients with DCM but decreased after bariatric surgery. As a predictor of cardiovascular disease, Hsp60 correlates with inflammatory markers and may be a molecular link between adipose tissue inflammation and the development of cardiovascular disease (Sell et al., 2017). TLR4 mediates HSP60-induced apoptosis in cardiac myocytes from WT mice, which appears to be involved in replacement fibrosis (Heiserman et al., 2015).

## 5 Diabetic kidney disease

Rising global prevalence of T2DM and chronic kidney disease (CKD) has prompted research into DKD (Anders et al., 2018). The leading cause of kidney failure worldwide is DKD (Reidy et al., 2014). More serious, All-cause mortality in DKD patients was 31% and 10-year mortality was 20% (Afkarian et al., 2013). Hyperglycemia is the main cause of the development of DKD. The earliest signs of DKD are microalbuminuria (>30 mg/day), which progresses to macroalbuminuria (>300 mg/day) and decreased glomerular filtration rate (GFR), culminating in end-stage renal disease (ESRD) (Rocco and Berns, 2009). Metabolic changes associated with T2DM can lead to glomerular hypertrophy, glomerulosclerosis, and tubulointerstitial inflammation and fibrosis (Tervaert et al., 2010). More than 40% of people with obesity and T2DM develop renal impairment as microvascular complications, and 30% go on to develop end-stage renal disease (Adler et al., 2003). Tight glycemic control significantly reduces the incidence of diabetic nephropathy, suggesting that hyperglycemia-induced metabolic changes, including changes in energy utilization and mitochondrial dysfunction, play a key role in disease development (Reidy et al., 2014).

### 5.1 Effects and underlying mechanisms of bariatric surgery in improving diabetic kidney disease

A large retrospective observational cohort study of patients with T2DM showed that bariatric surgery significantly reduced the incidence of kidney disease relative to nonsurgical interventions (6.4% vs. 14%) (O’Brien et al., 2018). For patients with pre-existing kidney disease, almost 80% improvement in albuminuria in the short and long term after bariatric surgery including RYGB and SG (Young et al., 2019). Obvious renal histopathological lesions including glomerulosclerosis, mesangial matrix expansion, and podocyte hypertrophy are prevalent in T2DM and can be ameliorated by bariatric surgery (Navarro-Díaz et al., 2006). This is consistent with the anti-proteinuric effect of SG and RYGB in STZ-induced DKD rats (Canney et al., 2020; Xiong et al., 2020). Meanwhile, improvements in glomerular structure and ultrastructure were also observed in animal models. An observational retrospective cohort study demonstrated that bariatric surgery, particularly RYGB surgery, significantly improved Estimated glomerular filtration rate (eGFR) for up to 3 years. Bariatric surgery should be considered for patients with DKD who have failed lifestyle changes and medical therapy, which have potential benefits in slowing DKD progression (Friedman and Wolfe, 2016). As a means to improve outcomes in patients with renal disease, bariatric surgery has myriad potential roles that require more clinically relevant studies to explore (Martin et al., 2020).

Bariatric surgery reduces weight and improves hypertension and hyperglycemia, which may explain some of the positive effects on albuminuria (Navarro-Díaz et al., 2006; Getty et al., 2012). Important mechanisms of DKD pathogenesis are advanced glycation end products and oxidative stress (Giacco and Brownlee, 2010). However, the role of inflammation is increasingly recognized as key to the progression of kidney disease (Fenske et al., 2013).

Chronic low-grade inflammation is often associated with T2DM (Sabatino et al., 2017). C-reactive protein (CRP) is associated with the development of DKD (McDonald et al., 2004; Rao, 2012). CRP promotes renal fibrosis and inflammation in a TGF-β-dependent manner (You et al., 2021). Bariatric surgery may reduce CRP and repair the pro-inflammatory chemokine and cytokine milieu, while enhancing anti-inflammatory chemokine and cytokine production (Illán-Gómez et al., 2012). CC-chemokine ligand 2 (CCL2), a protein closely associated with obesity and diabetic nephropathy (Tesch, 2008). Bariatric surgery-induced reduction in CCL-2-related inflammation is associated with improved renal function and reduced renal fibrosis (Bueter et al., 2010; Monte et al., 2012; Fenske et al., 2013; Wu et al., 2014). Similar improvement in urinary CCL-2/creatinine ratio at 4 weeks after RYGB and SG, but additional effect of RYGB in reducing chemokine ligand 18 (CCL-18) (Bueter et al., 2010). The AMPK/TGF-β pathway has been identified as a key pathway regulating inflammation and profibrosis in diabetic nephropathy. In a rodent kidney model, RYGB partially reversed DKD fibrosis by reducing TGF-β signaling (Vangoitsenhoven et al., 2020). At the same time, renal fibrosis was reversed after surgery, which may be related to the blockade of the renin-angiotensin system (Lambers Heerspink et al., 2013; Berney et al., 2021). Furthermore, several recent observations suggest that elevated endogenous gut hormones, such as GLP-1, may be an intermediate mediator of the anti-inflammatory effects of RYGB (Navaneethan et al., 2010).

## 6 Summary and future perspective

In recent years, bariatric surgery has grown as an emerging treatment for T2DM and related comorbidities. Currently, there is a growing body of strong evidence for the efficacy and safety of bariatric surgery (Arterburn et al., 2020). Therefore, for severely obese patients with T2DM, it is necessary to add bariatric surgery to the treatment decision-making option.

High-quality evidence from randomized controlled trials suggests that bariatric surgery is superior to medical therapy for enhancing glycemic control and achieving T2DM remission. The continued global prevalence of NAFLD, DCM and DKD as comorbidities of T2DM means that this is an important priority for healthcare and research. Bariatric surgery to treat these major T2DM comorbidities has obvious advantages over medical treatment in terms of duration and improvement.

In T2DM, fibrosis in various organs is driven in part by inflammation caused by IR itself. TGF-β is a common mediator of organ fibrosis in T2DM (Rockey et al., 2015). Unfortunately, the role of TGF-β in bariatric surgery ameliorating myocardial fibrosis has not yet been elucidated. A liver-specific fibrotic pathway involving TLR4 has also been reported in studies of cardiomyocyte fibrosis. It can be seen from these that the molecular systems related to T2DM-induced fibrosis are very extensive and complex, and need to be further explored and clarified.

Future research should focus on identifying key transcriptional, proteomic, and metabolomic changes that occur in vital organs of the body such as the heart, liver, and kidneys after bariatric surgery, which will help differentiate between the benefits and harms of bariatric surgery in people with T2DM impact and can inform the design of novel medical treatments that replicate the beneficial effects of surgery, while providing ideas for addressing the adverse consequences of surgery, thereby maximizing its therapeutic benefits.
